# Development of a Dynamic Network Model to Identify Temporal Patterns of Structural Malformations in Zebrafish Embryos Exposed to a Model Toxicant, Tris(4-chlorophenyl)methanol

**DOI:** 10.3390/jox13020021

**Published:** 2023-06-16

**Authors:** Ashley V. Schwartz, Karilyn E. Sant, Uduak Z. George

**Affiliations:** 1 Computational Science Research Center, San Diego State University, San Diego, CA 92182, USA; avschwartz@sdsu.edu; 2 Department of Mathematics and Statistics, San Diego State University, San Diego, CA 92182, USA; 3 School of Public Health, Division of Environmental Health, San Diego State University, San Diego, CA 92182, USA; ksant@sdsu.edu

**Keywords:** embryonic development, dynamic network model, embryonic morphology, toxicant, zebrafish

## Abstract

Embryogenesis is a well-coordinated process relying on precise cues and environmental signals that direct spatiotemporal embryonic patterning. Quite often, when one error in this process occurs, others tend to co-occur. We posit that investigating the co-occurrence of these abnormalities over time would yield additional information about the mode of toxicity for chemicals. Here, we use the environmental contaminant tris(4-chlorophenyl)methanol (TCPMOH) as a model toxicant to assess the relationship between exposures and co-occurrence of developmental abnormalities in zebrafish embryos. We propose a dynamic network modeling approach to study the co-occurrence of abnormalities, including pericardial edema, yolk sac edema, cranial malformation, spinal deformity, delayed/failed swim bladder inflation, and mortality induced by TCPMOH exposure. TCPMOH-exposed samples revealed increased abnormality co-occurrence when compared to controls. The abnormalities were represented as nodes in the dynamic network model. Abnormalities with high co-occurrence over time were identified using network centrality scores. We found that the temporal patterns of abnormality co-occurrence varied between exposure groups. In particular, the high TCPMOH exposure group experienced abnormality co-occurrence earlier than the low exposure group. The network model also revealed that pericardial and yolk sac edema are the most common critical nodes among all TCPMOH exposure levels, preceding further abnormalities. Overall, this study introduces a dynamic network model as a tool for assessing developmental toxicology, integrating structural and temporal features with a concentration response.

## 1. Introduction

Embryonic development is a rapid, highly coordinated process requiring precisely timed and patterned signals that direct cellular, tissue, and organ architecture. When these signals are dysregulated or poorly timed, adverse developmental effects can occur ranging from perturbed cell signaling and/or polarity to structural malformations or spontaneous abortion. Common modulators of these processes include diet, pharmaceuticals, or environmental exposures. While factors such as diet and medications can be at least somewhat controlled, ambient environmental or occupational exposures are challenging to prevent or mitigate.

The zebrafish (*Danio rerio*) is a widely used model for aquatic and embryonic development in toxicology studies [1,2,3]. Because they are vertebrates, their structural embryonic development is similar to humans and, thus, they can be used as both aquatic and human development models. Their genome is well characterized, and shares > 70% genetic homology with humans, allowing for the use of a wide array of molecular tools [4]. Perhaps the most advantageous characteristics of the zebrafish model are their external fertilization, large clutches of many embryos, and embryonic transparency allowing for direct visualizations of organ structures. Common teratogenic hallmarks of toxicological exposures include structural malformations, edemas, and functional or behavioral impairments.

Adverse developmental effects following toxicological exposure in the zebrafish model are widely studied to assess emerging toxicants. An interesting subset of these studies considers the co-occurrence of adverse effects during development [5,6]. Patterns in these co-occurring events reveal additional information about toxicant modes of action and adverse developmental behavior and are hypothesized to reveal predisposition to further health concerns. We aim to add to the understanding of co-occurring adverse developmental events in the zebrafish model using a model toxicant.

Tris(4-chlorophenyl)methanol (TCPMOH) is an environmental pollutant, considered a contaminant of emerging concern. TCPMOH is a halogenated organic compound, believed to be lipophilic, highly persistent in the environment, and bioaccumulative [7]. It has been detected in coastal wildlife samples as well as human samples, including breast milk [8,9,10,11]. The source of TCPMOH is still relatively unknown but its structural similarity to, and co-occurrence with, the legacy insecticide dichlorodiphenyltrichloroethane (DDT) raises concern and solidifies the need for toxicity assessment [8,12].

We have previously shown the hazard posed by TCPMOH exposures in the zebrafish, examining changes in gene expression and prevalence of common structural deformities and impairments [13]. We reported mortality, prevalence of swim bladder inflation, and embryonic malformations for five experimental groups: 0.01% *v*/*v* DMSO (control), 0.1 µM, 0.5 µM, 1 µM, and 5 µM TCPMOH. Mortality increased in a concentration- and time-dependent manner throughout the developmental period. In the previous work, we excluded animals exposed to 5 µM TCPMOH due to high mortality that begins as early as 2 dpf. Significant changes in morphology began as early as 4 dpf in the most highly exposed group (1 µM), including pericardial edema, yolk edema, craniofacial malformations, and swim bladder inflation. Embryos exposed to 0.1 µM TCPMOH were largely unaffected by exposure, with statistically significant changes from controls only occurring for swim bladder inflation. It was concluded that TCMPOH increases the incidence of embryonic abnormalities and health outcomes such as pericardial edema, craniofacial malformations, impaired swim bladder inflation, and even mortality. Though co-occurrence of these effects, especially pericardial and yolk sac edema, was noted, the effect and temporality of one morphology on the other is poorly understood or characterized.

To investigate the co-occurrence and relationships between abnormalities observed in TCPMOH exposed samples over time, we consider the methodology of dynamic networks. Networks are widely used in the field of biology to explain the interactions and patterns between elements of a system [14]. Applications include metabolic networks [15], protein networks [16,17,18], food networks [19], and gene expression networks [20,21], among many others. A network is composed of nodes that represent the elements of a system and links that describe the interactions/associations among them. The complexity of the network is associated with the number of nodes and links within the model and can increase greatly depending on the application and the number of parameters within the model. For the application at hand, nodes represent developmental abnormalities and links represent associations among these abnormalities. The co-occurrence/association between two abnormalities at any given time is high if both co-occur at that time. The nodes of the network are held fixed for all time and exposure groups but the links between the nodes are allowed to change based on temporal changes in associations among the abnormalities. Hence, the network is referred to as a dynamic network model. The network model is used to extract key information about: (i) the temporal patterns of co-occurrence of TCPMOH-induced developmental abnormalities, and (ii) the temporal difference in network behavior/connectivity for varying exposure levels.

Despite its potential to further our understanding of the co-occurrence of multiple adverse outcomes, there are no reports of the use of dynamic network models to associate and examine morphological changes during zebrafish embryonic development. The dynamic network model captures the patterns in abnormality co-occurrence over discrete time under varying exposure levels. Furthermore, this study introduces a tool in the evaluation of developmental toxicity and teratology by using dynamic networks, drawing upon not only reductionist (single endpoint) results but also co-occurrence of multiple adverse outcomes. Here, we use this dynamic network model to characterize the response to TCPMOH in zebrafish, though this strategy is widely applicable to developmental toxicology research.

## 2. Materials and Methods

### 2.1. Chemicals

Tris(4-chlorophenyl)methanol (TCPMOH; CAS #3010–80–8, 95% purity) was purchased from Alfa Aesar (Ward Hill, MA, USA), and dimethyl sulfoxide (DMSO) was purchased from Fisher Scientific (Pittsburgh, PA, USA). The chemical structure and formula for TCPMOH is shown in Figure S1. Concentrated (10,000×) stock solutions of TCPMOH [1–50 mM] for embryonic exposures were prepared in DMSO and stored at room temperature in amber glass vials away from light until use. All experimental procedures involving TCPMOH were performed using appropriate safety precautions.

### 2.2. Zebrafish Husbandry & Care

Wild-type (AB) strain zebrafish were housed in an automated Aquaneering zebrafish system at San Diego State University in the Toxicology Laboratory. Water temperature was maintained at 28.5 °C, pH 7.2–7.3, conductivity 650–750 µS, and light cycling was maintained at a 12:12 light:dark cycle. Fish were provided the recommended amount of GEMMA Micro 300 powdered diet once daily (Skretting; Westbrook, ME, USA). Nitrates, nitrites, ammonia, and chlorine were measured weekly. Breeding tank populations contained 15–20 adult fish (2:3 male:female ratio). All animal use protocols have been approved by the San Diego State University Institutional Animal Care & Use Committee and meet or exceed all recommended practices for zebrafish care (PHS Assurance Number 16–00430).

Embryos were collected from breeding tanks between 0 and 1 h post-fertilization (hpf), washed, and housed in clean polystyrene dishes containing 0.3X Danieau’s medium (17 mM NaCl, 2 mM KCl, 0.12 mM MgSO_4_, 1.8 mM Ca(NO_3_)_2_, 1.5 mM HEPES, pH 7.6). At 6–8 hpf, embryos were sorted for viability and quality, and embryos from different breeding tanks were consolidated and then randomized into clean 100 mm polystyrene petri dishes with fresh 0.3X Danieau’s medium and incubated at 28.5 °C overnight on a 12:12 h light:dark cycle.

### 2.3. Exposures

At 1 day post fertilization (dpf), embryos were manually dechorionated using watchmaker’s forceps and individually transferred into wells of a polystyrene 24-well plate containing 1 mL of 0.3X Danieau’s medium supplemented with 0.01% *v*/*v* dimethyl sulfoxide (DMSO) (vehicle control), 0.5 μM, 1 μM, or 5 μM TCPMOH (n = 20–38 embryos per group). Exposure media were refreshed daily to prevent hypoxia throughout the study. Individual housing allowed for time-series data of each embryo throughout the study.

### 2.4. Microscopy

Zebrafish embryos were individually imaged daily from 1–7 dpf to observe the developmental process in vivo. To immobilize embryos and larvae for live imaging, fish were briefly anaesthetized using MS-222 (2% *v*/*v*) and gently transferred into droplets of 3% methylcellulose on optical slides. All imaging was performed using a Nikon Ti-2 inverted microscope. Brightfield images were acquired at 20× and 40× magnification. Following imaging, each fish was rinsed briefly to remove methylcellulose and allowed to recover in fresh 0.3X Danieau’s medium before being transferred back to well plates for repeated measures.

### 2.5. Quantitative Analysis of Embryonic Morphology

**Figure 1:**
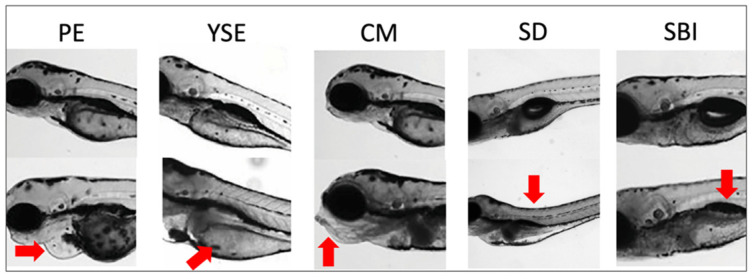
Examples of deformities and impairments observed following TCPMOH exposures. Abnormalities recorded in the zebrafish include pericardial edema (PE), yolk sac edema (YSE), cranial malformations (CM), spinal deformities (SD), and delayed/failed swim bladder inflation (SBI). Mortality is also assessed but no figure is shown. Red arrows point to the specified abnormalities.

The prevalence (binary yes/no assessment) of common embryonic morphologies and impairments was recorded daily for each fish. Specifically, pericardial edema (PE), yolk sac edema (YSE), craniofacial malformations (CM), and spinal deformities (SD) were quantified. Mortality (M) and delayed or failed swim bladder inflation (SBI) were also recorded (Figure 1). Swim bladder inflation is considered delayed if it is not inflated by 4 dpf, the time at which the majority of control group swim bladders are inflated. Collectively, these aberrant morphologies and states are herein referred to as “abnormalities”. The incidence of developmental abnormalities was 2–7 dpf for each exposure group (Table S1), which has been previously published [13]. This study aims to expand on the previously published work by utilizing a methodology to analyze the dynamic co-occurrence of the observed abnormalities.

### 2.6. Abnormality Co-Occurrence

**Figure 2:**
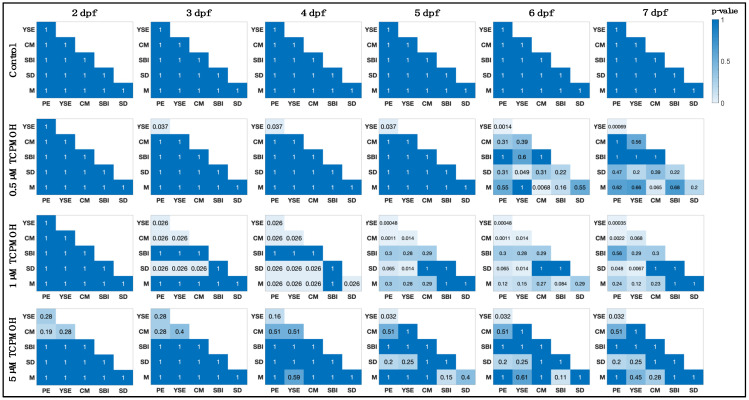
Heat maps of association for exposure groups across time. Heat maps of *p*-values computed from Fisher’s exact test. Association significance is computed and reported for each abnormality pair assessed during the developmental stage for each exposure group. Abnormalities include pericardial edema (PE), yolk sac edema (YSE), cranial malformations (CM), spinal deformities (SD), delayed/failed swim bladder inflation (SBI), and mortality (M).

The intensity of abnormality co-occurrence each day post fertilization (referred to here as ‘abnormality associations’) was computed using Fisher’s exact test (Figure 2) for each combination pair of binary abnormality outcomes. Statistical testing was computed in the MATLAB v.2022a programming language using the Statistics and Machine Learning Toolbox (v. 12.3). The MATLAB programming language allows for the automation of association calculations among the large set of parameter combinations. The resulting *p*-values are reported as heatmaps in Figure 2, where lighter colors represent a stronger significance of abnormality association.

Each sample group at 2 dpf has low co-occurrence significance, with most reported *p*-values being 1. As time continues, there is a general increase in the association between abnormalities for exposure groups, with many *p*-values reported as <0.05. Figure 2 demonstrates the differing abnormality co-occurrence response among time points and exposure groups and between outcome pairs. The control group did not present with associations between abnormalities (*p* = 1) during the developmental process. We infer from Figure 2 that abnormality co-occurrence is concentration- and time-dependent and utilize this knowledge to build a framework for extracting patterns in abnormality co-occurrence.

### 2.7. Dynamic Network Model Describes the Temporal Patterns of Embryonic Abnormality Co-Occurrences

**Figure 3:**
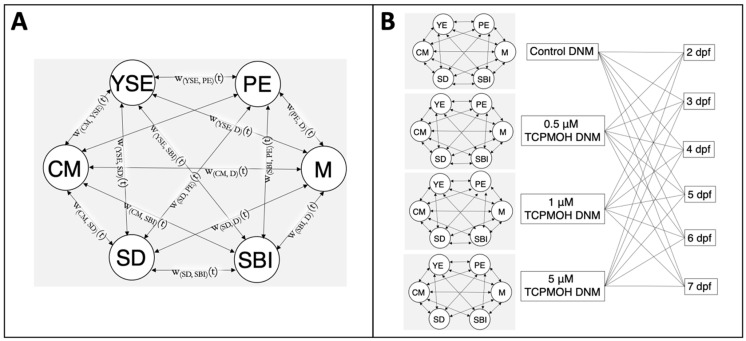
Dynamic network model (DNM) structure. The dynamic network model connecting the observed abnormalities using association links over time. Abnormalities include pericardial edema (PE), yolk sac edema (YSE), cranial malformations (CM), spinal deformities (SD), delayed/failed swim bladder inflation (SBI), and mortality (M). (**A**) An enlarged version of the network demonstrating the time-dependent weighted links that exist in the networks. Weighted links are assigned using the co-occurrence *p*-values reported in Figure 2. (**B**) A complete illustration of the network models for each exposure group that are configured for each time point under consideration.

Dynamic networks are graphs that represent the relationships between nodes over time. The proposed dynamic network model to investigate abnormality co-occurrence is presented (Figure 3), where nodes represent observed abnormalities and links represent the associations between them. The observed embryonic abnormalities include pericardial edema, yolk sac edema, craniofacial malformations, spinal deformities, delayed swim bladder inflation, and mortality. The network connections (links) between nodes are assigned weights of association indicating the strength of the co-occurrence [22,23,24]. Weights are computed utilizing the significance of association from Fisher’s exact test as reported in Figure 2. As the association between abnormalities within the model varies with time, we configure a dynamic weighted network model for each exposure level. In total there are four distinct network models each with 90 links over the dynamic time span, or 360 in total. Each network is fully determined by its adjacency matrix, which is used to calculate network properties and topology. These matrix representations are developed as follows. The formal mathematical graph representation of the network, as described in [25], is G(t)={N(t), L(t), f(t)}, with time t, nodes N, links L, and mapping function f:N×N that connects node pairs for network topology. The proposed network model contains K=6 nodes and *M =* 15 weighted links. The nodes $N\left(t\right)=\left[n_{1}\left(t\right),n_{2}\left(t\right)\ldots ,\;n_{K}\left(t\right)\right]$ and the links $L\left(t\right)=\left[l_{1}\left(t\right),\;l_{2}\left(t\right),\;\ldots ,\;l_{M}\left(t\right)\right]$. The weights of the links $w_{i,j}\left(t\right)$ between nodes $n_{i}$ and $n_{j}$ for i,j=1,…6 is computed as $w_{ij}\left(t\right)=100*\left(1-p_{ij}\left(t\right)\right)$ where $p_{ij}\left(t\right)$ is the *p*-value of association between nodes $n_{i}$ and $n_{j}$ at time t. This ensures that a statistically significant result (*p* < 0.05) is given a large weight (>95%) within the model. The links are undirected, and the weights satisfy that $w_{ij}\left(t\right)=w_{ji}\left(t\right)$.

The mapping function f(t):N(t)×N(t) is the adjacency matrix, denoted A(t) that indicates if node i is connected to node j and at what magnitude. A(t) is a N×N matrix with elements $A_{ij}\left(t\right)$ such that

(1) $$A_{ij}\left(t\right)=\{\begin{array}{ll} w_{ij}\left(t\right) & \mathrm{if}\;\mathrm{there}\;\mathrm{is}\;\mathrm{an}\;\mathrm{edge}\;\mathrm{between}\;\mathrm{nodes}\;i\;\mathrm{and}\;j\;\mathrm{at}\;\mathrm{time}\;t\\ 0 & \mathrm{otherwise}. \end{array}$$

A(t) is a symmetric matrix since $w_{ij}\left(t\right)=w_{ji}\left(t\right)$.

Each dynamic network model for all exposure groups is fully determined by its matrix A(t), which contains the strength of co-occurrence between any two nodes at time t. The dynamic weighted networks in adjacency matrix representation have been developed in MATLAB 2022a. Network properties and topology are extracted by analyzing A(t) over time for each exposure group. The analysis, referred to as centrality analysis, can identify the strength of connections between all nodes in the network and the nodes with the greatest influence on the network. In other words, centrality analysis can identify the magnitude of abnormality co-occurrence over time for each exposure group and the critical abnormalities influencing this co-occurrence.

### 2.8. Network Centrality Analysis

Centrality is a score given to a network that assigns a quantitative value for the co-occurrence between observed abnormalities for differing exposure groups over time. Centrality scores can be computed in a variety of different ways and are often application-dependent. Here, degree centrality and eigenvector centrality are utilized to investigate the temporal pattern of structural abnormality co-occurrence both globally (i.e., the differences in abnormality co-occurrence between exposure groups) and on an individual node basis (i.e., individual node impact on the connectivity/co-occurrence of abnormalities in a network over time).

Degree centrality of an abnormality characterizes the strength of its co-occurrence with other abnormalities within the network. Formally, the degree of a node at time t, denoted as $d_{K}\left(t\right)$ for node $n_{K}\left(t\right)$, in a weighted network is the sum of all weighed links forming connections to the node [26]. Formally, we have $d_{K}\left(t\right)=\underset{j\neq i}{\sum }A_{ij}\left(t\right)$. A node k that has a high degree centrality score at a given time t represents an abnormality/node that has a high connectivity/co-occurrence with other structural abnormalities in the network at that given time. The global centrality score of an exposure group’s network at time t is defined as the maximum degree centrality score at that time (i.e., $max(d_{1}\left(t\right),\;\ldots ,d_{K}\left(t\right)),\;K=6$). The degree centrality score for each individual node, or abnormality, is computed and the maximum degree score is utilized for global centrality analysis.

Eigenvector centrality is an extension of degree centrality. It considers both individual node importance (i.e., weight of immediate connections) and the importance of its neighbors [14]. For example, if a node is connected to an influential node, its own centrality score will increase. This is often referred to as transitive influence. The eigenvector centrality of a node is measured using the eigenvalues λ of the adjacency matrix A(t) and is computed from det[A(t)−λ I]=0, where I is the identity matrix and det[·] the determinant. The maximum eigenvalue (i.e., spectral radius) denoted ρ=max{λ}, reveals the node corresponding to the maximum transitive influence on the network.

Degree and eigenvector centrality are computed in MATLAB 2022a, according to the formulas described here on the adjacency matrices developed in Section 2.7. A global centrality analysis utilizing both the maximum degree and the spectral radius is utilized to determine the network connectivity differences between exposure groups. Individual node centrality analysis, using both degree and eigenvector centrality, is utilized to determine individual node impact on the connectivity of the networks over time.

## 3. Results

### 3.1. Global Network Centrality Reveals TCPMOH’s Impact on the Temporal Pattern of Structural Abnormality Co-Occurrence

**Figure 4:**
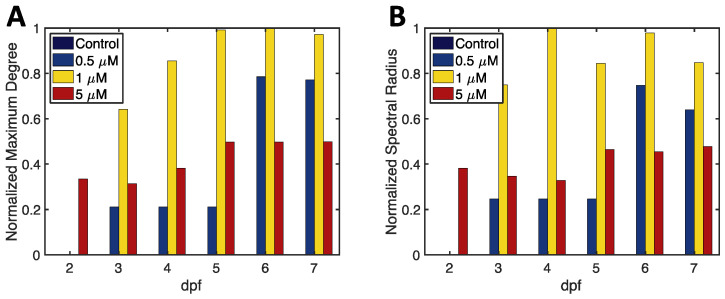
Complete network centrality scores for the dynamic network model. (**A**) The normalized maximum degree centrality score for all exposure groups each day post fertilization. (**B**) The normalized spectral radius centrality score for all exposure groups each day post fertilization. Centrality scores of zero occur for the control group at all time points and for 0.5 μM and 1 μM at 2 dpf.

Global network degree and eigenvector centrality scores were used to investigate the differences in abnormality co-occurrence between exposure groups. The maximum degree over time for each exposure group is shown in Figure 4A, where a larger maximum degree indicates a higher significance of co-occurrence between abnormalities. The magnitude of degree centrality varies between TCPMOH exposure levels, revealing differences in abnormality co-occurrence. The 1 μM TCPMOH group maximum degree is the largest in magnitude, from 3 dpf to 7 dpf, signifying the greatest amount of abnormality co-occurrence among exposure levels. There is no abnormality co-occurrence in the control group as the maximum degree is zero at each time point. Interestingly, the 5 μM TCPMOH group is the only group with abnormality co-occurrence at 2 dpf, with the magnitude of degree centrality remaining smaller than the 1 μM TCPMOH group for the remainder of the developmental process. This is likely due to high incidence of abnormalities leading to early mortality. Global degree centrality analysis reveals that the rate of abnormality co-occurrence is greatest for the 1 μM TCPMOH group.

The spectral radius over time for each exposure group is shown in Figure 4B. The control group’s spectral radius is zero at all time points, while the 1 μM exposure group is the greatest in magnitude at 3–7 dpf. Global eigenvector centrality therefore reveals that the 1 μM exposure group contains the greatest rate of abnormality co-occurrence while including direct and transitive influence.

Degree and eigenvector centrality revealed similar patterns between exposure groups. The 5 μM exposure group contained abnormality co-occurrence as early as 2 dpf with the magnitude of co-occurrence staying constant beyond 5 dpf. The 1 μM exposure group had the greatest connectivity and therefore the strongest co-occurrence between abnormalities.

### 3.2. Individual Node Centrality Analysis Reveals Yolk and Pericardial Edema as the Predominant Nodes among All Exposure Levels Most Associated with Further Abnormalities

Morphological endpoints that are highly correlated and influential to (preceding) others may reveal essential modes of action for a chemical and suggest potential pathways of toxicity. To investigate the abnormality that has the most influence on the incidence of other observed abnormalities throughout the developmental process, individual node centrality analysis was used. Nodes/abnormalities with a high centrality magnitude are those that co-occur with remaining assessed abnormalities.

The critical nodes corresponding to the node with the maximum degree and spectral radius are reported in Table 1. Critical abnormalities include pericardial edema, yolk sac edema, spinal deformity, and cranial malformation. Interestingly, pericardial edema and yolk sac edema had equivalent centrality scores 3–5 days post-fertilization for the 0.5 μM exposure group, demonstrating their co-occurrence with one another. Beyond 5 dpf, spinal deformity is the abnormality with the highest rate of co-occurrence in the 0.5 μM exposure group. Yolk sac edema is the critical abnormality for the 1 μM exposure group 5–7 days post-fertilization, with earlier time points having no centrality around a particular node. Degree centrality maximum score and spectral radius score revealed similar critical abnormalities for 0.5 μM and 1 μM exposure groups for different dpf. The critical abnormality for the 5 μM exposure group was identical for both centrality scores each day, except at 7 dpf. The observed difference in critical abnormality may have occurred because the spectral radius captures both the weight of immediate connections and the importance of its neighboring nodes, while the degree centrality maximum score captures only the weight of immediate connections. The spectral radius score is higher than the degree centrality maximum score for pericardial edema at 7 dpf and, therefore, can infer that compared to yolk sac edema, pericardial edema is connected to more influential nodes at 7 dpf. In the 5 μM exposure group, pericardial edema and cranial malformation co-occurred at the highest rate at 2 dpf and the network was central around either pericardial or yolk sac edema for the remaining days.

Unique maximum node importance for any time point or exposure group is assigned to pericardial edema, yolk sac edema, and spinal deformity, for 35%, 45%, and 20% of the time, respectively (Table 1). Maximum node importance (either unique or tie) for all time points and exposure groups is assigned to pericardial edema, yolk sac edema, spinal deformity, and cranial malformation for 42%, 42%, 11%, and 5% of the time, respectively (Table 1). Pericardial and yolk sac edema make up 80% of the reported central nodes for all exposure networks combined.

**Figure 5:**
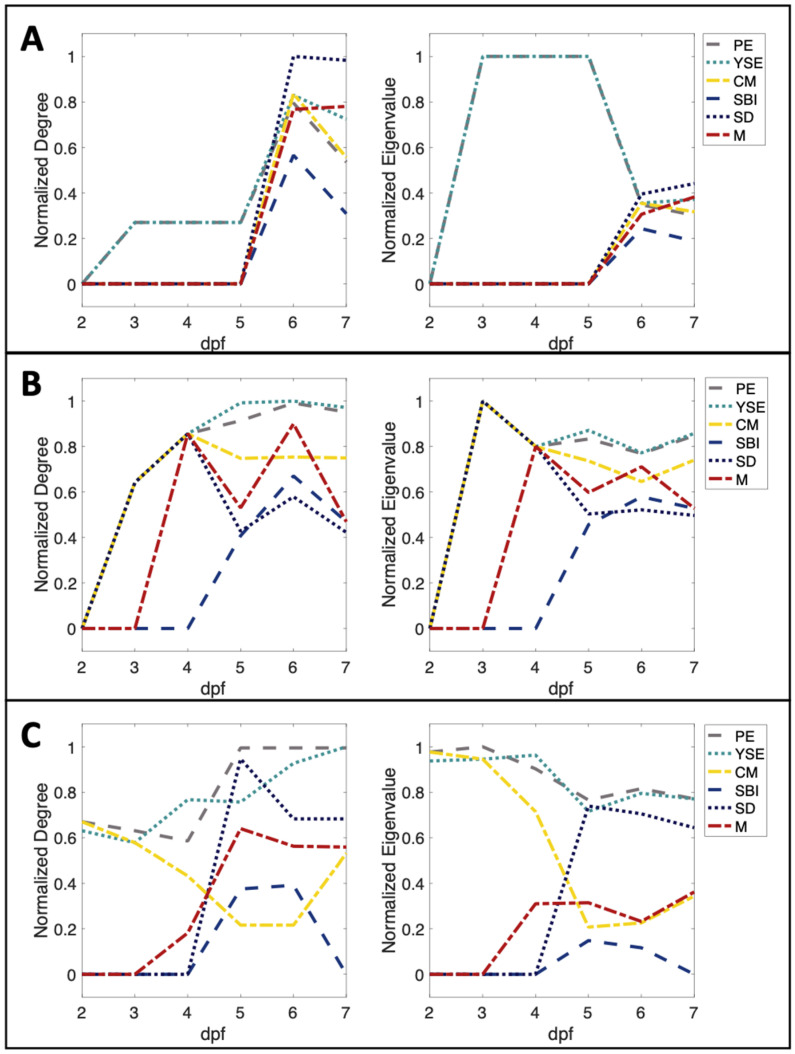
Individual node centrality scores over time. Individual node degree and eigenvector centrality scores over time for (**A**) 0.5 μM TCPMOH, (**B**) 1 μM TCPMOH, and (**C**) 5 μM TCPMOH. The degree and eigenvector centrality scores are computed for each node in the dynamic network model. Nodes represent abnormalities assessed: pericardial edema (PE), yolk sac edema (YSE), cranial malformations (CM), spinal deformities (SD), delayed/failed swim bladder inflation (SBI), and mortality (M). The control group is omitted as centrality scores are zero for each node across all time points.

Node centrality measures over time are reported in Figure 5. The centrality score for each node within the model varies dynamically, with the most influential node varying as described previously. Pericardial edema, yolk sac edema, and cranial malformations are the first abnormalities to present with co-occurrence influence for the 5 μM exposure group at 2 dpf. Interestingly, among all exposure groups, pericardial edema and yolk sac edema followed similar trends throughout the developmental stage. In the 0.5 μM exposure group, the centrality scores were equal for pericardial edema and yolk sac edema until 7 dpf.

**Figure 6:**
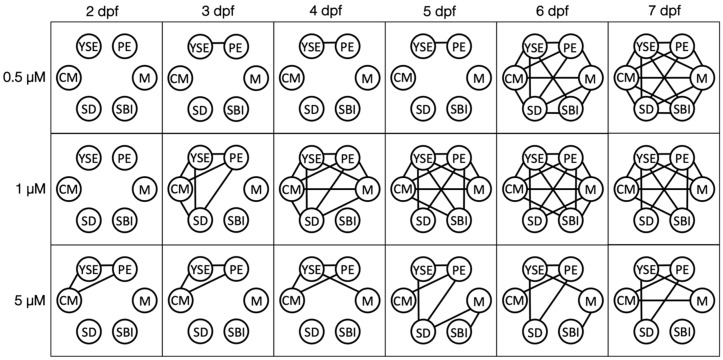
Network connectivity over time. Individual networks over time for each exposure group demonstrate the abnormalities (nodes) and their co-occurrence (links). Nodes represent abnormalities assessed: pericardial edema (PE), yolk sac edema (YSE), cranial malformations (CM), spinal deformities (SD), delayed/failed swim bladder inflation (SBI), and mortality (M). The control group is omitted as there is no abnormality co-occurrence across all time points. Links are present if the two abnormalities co-occur at the given time point.

Individual node centrality scores can be investigated further by analyzing the temporal changes in network connectivity as seen in Figure 6. In all cases, when co-occurrence was present between any abnormalities, the link between pericardial and yolk sac edema was also present. The continual co-occurrence of these abnormalities signifies the role they play in the incidence of further abnormalities over time. The strength of their influence is supported by their similar centrality scores over time in all exposure groups, as seen in Figure 5.

The role of pericardial and yolk sac edema is especially apparent in the 0.5 μM group. The 0.5 μM group presents with the co-occurrence of pericardial and yolk sac edema at early points: 3, 4, and 5 dpf. At 6 dpf, there is a striking increase of co-occurrence in abnormalities jumping from one network link at 5 dpf to 11 at 6 dpf. Interestingly, the critical node, or abnormality co-occurring at the highest rate to other abnormalities, is spinal deformity at 6 and 7 dpf, while the critical nodes at 3, 4, and 5 dpf are jointly pericardial and yolk sac edema. The change in critical node is due to the co-occurrence of spinal deformity and mortality at 6 dpf. The early presence and co-occurrence of pericardial and yolk sac edema may be causing incidences of further deformities, as both abnormalities continue to co-occur with one another and the incidence of remaining abnormalities is at a high rate.

## 4. Discussion

Zebrafish are widely used to investigate developmental toxicity [3,27,28,29]. Morphological endpoints, such as those parameters (abnormalities) described here, are often reported and analyzed as individually occurring abnormalities at a single time point. However, common mechanisms of teratogenesis govern many developmental processes in different organ systems. Moreover, we posit that investigating the co-occurrence of these abnormalities over time would yield additional information about the mode of toxicity for chemicals. The plethora of data generated in toxicology studies can be complex to analyze and represent in meaningful simple ways. The goal of this study was to develop and apply a methodology to assess toxicity by incorporating the co-occurrence of abnormalities observed. The methodology of network models has been utilized in the field of biology for decades [30] and we introduce here, to our knowledge, the first dynamic network model for morphological toxicity assessment in zebrafish.

We have succinctly represented six developmental abnormalities occurring in the zebrafish and their associations across 2–7 dpf using a dynamic network model. Network science and analysis signified the differences in abnormality associations between exposure groups. TCPMOH-exposed samples displayed an increase in embryonic abnormality co-occurrence across all exposure levels in comparison with the control group as measured by centrality scores maximum degree (Figure 4A) and SR (Figure 4B). The control group displayed zero node importance within the model, or zero abnormality co-occurrence, as expected. The maximum degree and SR are reported as a nonlinear concentration response. We see the 1 μM TCPMOH exposure level has greater maximum degree and SR scores among nearly all time points.

One observation of note is the decreased abnormality association scores for embryos and larvae in the 5 µM exposure group compared to those in the 1 µM group. This is likely an artifact of survival bias, since mortality was significantly increased as early as 2 dpf in the 5 µM exposure group (Table S1). The inclusion of mortality in the network model is utilized to assess the temporal aspect of abnormality co-occurrence and its relationship to mortality at later time points. The early occurrence of mortality leads to survival bias because if mortality occurs, no other abnormalities could be assessed. In contrast, if mortality occurred at later time points, there is the opportunity for abnormality development prior to mortality. In the latter case, individual node centrality may be used to investigate the relationship between abnormality co-occurrence and mortality as an important toxicological endpoint. Therefore, we conclude that these network methods are most effective and accurate for sublethal exposures in early development.

Individual node centrality analysis indicates the importance of certain abnormalities when compared to others. Pericardial and yolk sac edema were the abnormalities most likely to co-occur with the remaining abnormalities in the model (Figure 5). The occurrence of cranial malformations, spinal deformities, delayed swim bladder inflation, and mortality were all closely associated with pericardial and yolk sac edema as measured by centrality, likely due to increased incidence of pericardial and yolk sac edema, but also because pericardial and yolk sac edema frequently precede these other abnormalities. An interesting finding was the identical centrality scores between pericardial and yolk sac edema in the 0.5 μM TCPMOH exposure group at early time points, which later switches to unique importance for spinal deformity. This could be pointing to the impact of early incidence of pericardial and yolk sac edema and how it relates to the onset of other abnormalities, including mortality, at later time points.

The role of pericardial and yolk sac edema in the temporal patterns in abnormality co-occurrence is further demonstrated by network connectivity (Figure 6). All cases of abnormality co-occurrence contained the connection between pericardial and yolk sac edema. Not only do the two abnormalities co-occur together at high rates, but they may be influencing the co-occurrence of further abnormalities over time, since they are typically the first to appear. These abnormalities frequently co-occur in conditions such as “blue sac disease” in fish, which is often incurred following exposures to polycyclic aromatic hydrocarbons and other organochlorines, including dioxins and polychlorinated biphenyls. More investigation with a diverse group of chemicals is needed to further test our model and determine this relationship, and this can provide insight into the biochemical mechanisms of disease.

The new methodology for abnormality co-occurrence analysis described here further supports and complements our most recently published work, which first characterized the individual incidence of abnormalities following embryonic TCPMOH exposures in zebrafish [13]. TCPMOH is an understudied environmental contaminant, becoming increasingly detected in environmental and biological samples with improvements in analytical methodologies [8,31,32,33,34,35]. Furthermore, TCPMOH has been detected in human adipose tissue and breast milk, and transplacental transfer has been confirmed in marine mammals [10,11,36,37]. Though often measured in biological matrices at similar concentrations to the legacy high-priority contaminant DDT and its metabolites, little is known about the toxicological consequences of these exposures. In our study, the concentrations utilized are likely supra-environmental, in order to capture a range of concentrations with teratogenic impacts, though this is somewhat speculative due to the limited data available on the environmental abundance of TCPMOH. However, TCPMOH is believed to bioaccumulate and biomagnify, as concentrations in biological matrices tend to be found in the ppb and ppt range. While the gaps in environmental and biological monitoring are evident, identification of concentration-response values for developmental toxicity is needed to contextualize risk. For these reasons, the uncovered developmental toxicity of this emerging compound is important to inform risk in future monitoring studies, and our network model can suggest potential mechanisms of toxicity for exploration.

The presented network model is scalable, and the number of abnormalities (nodes) that can be incorporated is unlimited. As we further test the rigor of this model with additional compounds, more subtle phenotypes or biochemical changes may be included as well. For example, measures of biomarkers, chemical metabolites, or dietary nutrients could be integrated into the model, along with malformations, to understand the role that metabolism and biochemical pathways may play in abnormal morphologies. Measurements such as centrality, as used in this model with multiple assay timepoints, can help to identify subtle changes that may precede a more deleterious abnormality or deformity, and enable the predictive use of this model. Ultimately, the expansion of these more subtle molecular and biochemical precedents in this model may be used to construct an adverse outcome pathway for distinct birth defects and adverse developmental phenotypes.

As the investigation of new chemicals discovered in our environment continues, the data generation and analysis of chemical toxicity is imperative. New methodologies are required to answer questions not yet answered. This study demonstrates the use of dynamic network modeling for abnormality co-occurrence analysis in zebrafish toxicity studies for the first time. The methodology described here is in no way limited to zebrafish morphology and may also be extended to any toxicity endpoint in question, including indices for mammalian development, rodent whole embryo culture, cancer metastasis, and more [38,39]. Singular time point analysis, in comparison to the dynamic time frame reported here, may also be done to determine toxicity parameters driving the process at hand. We hope to demonstrate the simplicity of representing large amounts of data into succinct networks to further inspire future research questions and directions for the toxicological assessment of environmental contaminants.

## Figures and Tables

**Table 1:** Most influential nodes in the dynamic network model. Nodes reported correspond to the maximum degree and spectral radius of the network model for each exposure group 2–7 days post-fertilization. Ties are reported if two nodes had the same centrality score. Ties with three or more nodes are omitted. The Control group is not reported as no nodes had centrality scores. Nodes represent abnormalities assessed: pericardial edema (PE), yolk sac edema (YSE), cranial malformations (CM), spinal deformities (SD), delayed/failed swim bladder inflation (SBI), and mortality (M).

**Degree Centrality Maximum Score**						
	**2 dpf**	**3 dpf**	**4 dpf**	**5 dpf**	**6 dpf**	**7 dpf**
0.5 μM TCPMOH	-	PE, YSE	PE, YSE	PE, YSE	SD	SD
1 μM TCPMOH	-	-	-	YSE	YSE	YSE
5 μM TCPMOH	PE, CM	PE	YSE	PE	PE	YSE
**Spectral Radius Score**						
	**2 dpf**	**3 dpf**	**4 dpf**	**5 dpf**	**6 dpf**	**7 dpf**
0.5 μM TCPMOH	-	PE, YSE	PE, YSE	PE, YSE	SD	SD
1 μM TCPMOH	-	-	-	YSE	YSE	YSE
5 μM TCPMOH	PE, CM	PE	YSE	PE	PE	PE

## Data Availability

Experimental (primary) data used in this manuscript is published and can be viewed at [13]. Additional information is available upon request.

## Supplementary Materials

The following supporting information can be downloaded at: https://www.mdpi.com/article/10.3390/jox13020021/s1, Table S1: Percent incidence of developmental deformities for each exposure group 2–7 days post-fertilization. Figure S1: Molecular structure of TCPMOH.
