# Supplementary material for: Development of a Dynamic Network Model to Identify Temporal Patterns of Structural Malformations in Zebrafish Embryos Exposed to a Model Toxicant, Tris(4-chlorophenyl)methanol
Source: J Xenobiot. 2023 Jun 16;13(2):284–97. doi: 10.3390/jox13020021 (PMC10301205; doi:10.3390/jox13020021)
Supplement: Supplementary file 1 [file jox-13-00021-s001.zip › jox-2392959-supplementary.pdf]

Table S1. Percent incidence of developmental deformities for each exposure group 2-7 days post-fertilization.

**Control**						
	**2 dpf**	**3 dpf**	**4 dpf**	**5 dpf**	**6 dpf**	**7 dpf**
Pericardial Edema	0	0	0	0	0	0
Yolk Sack Edema	0	0	0	0	0	0
Cranial Malformation	0	0	0	0	0	0
Spinal Deformity	0	0	0	0	0	0
Delayed/Failed Swim Bladder Inflation	0	0	54	29	25	25
Mortality	0	0	0	0	0	0
**0.5 µM TCPMOH**						
	**2 dpf**	**3 dpf**	**4 dpf**	**5 dpf**	**6 dpf**	**7 dpf**
Pericardial Edema	0	5	5	5	15	25
Yolk Sack Edema	5	5	5	5	20	8
Cranial Malformation	0	0	0	10	15	20
Spinal Deformity	0	0	0	5	15	15
Delayed/Failed Swim Bladder Inflation	0	0	60	35	35	35
Mortality	0	0	0	5	30	40
**1 µM TCPMOH**						
	**2 dpf**	**3 dpf**	**4 dpf**	**5 dpf**	**6 dpf**	**7 dpf**
Pericardial Edema	0	3	3	26	29	29
Yolk Sack Edema	3	3	3	32	37	37
Cranial Malformation	0	3	3	11	32	32
Spinal Deformity	0	3	3	11	11	11
Delayed/Failed Swim Bladder Inflation	0	0	95	89	89	89
Mortality	0	0	3	26	68	89
**5 µM TCPMOH**						
	**2 dpf**	**3 dpf**	**4 dpf**	**5 dpf**	**6 dpf**	**7 dpf**
Pericardial Edema	10	10	20	20	20	20
Yolk Sack Edema	15	15	20	25	25	25
Cranial Malformation	15	15	15	15	15	15
Spinal Deformity	0	0	0	5	5	5
Delayed/Failed Swim Bladder Inflation	0	0	100	90	90	90
Mortality	5	15	35	60	65	90

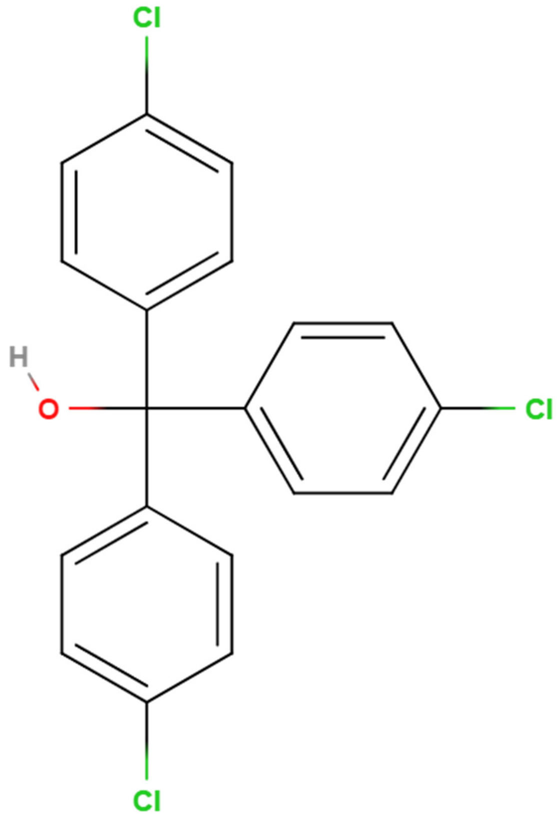

Figure S1. Molecular structure of TCPMOH. The chemical formula for TCPMOH is C19H15Cl3O (365.686 g/mol).
